# Updates of the In‐Gel Digestion Method for Protein Analysis by Mass Spectrometry

**DOI:** 10.1002/pmic.201800236

**Published:** 2018-11-25

**Authors:** Jennifer K. Goodman, Cleidiane G. Zampronio, Alexandra M. E. Jones, Juan R. Hernandez‐Fernaud

**Affiliations:** ^1^ School of Life Sciences University of Warwick Gibbet Hill Road Coventry CV 4 7AL UK; ^2^ Proteomics Research Technology Platform University of Warwick Gibbet Hill Road Coventry CV 4 7AL UK

**Keywords:** in‐gel digestion,mass spectrometry, proteomics, trypsin

## Abstract

The in‐gel digestion of proteins for analysis by liquid chromatograph mass spectrometry has been used since the early 1990s. Although several improvements have contributed to increasing the quality of the data obtained, many recent publications still use sub‐optimal approaches. Updates of the in‐gel digestion protocol has been presented in the study. It has been shown that alternative reducing, alkylating agent reactions, and tryptic digestion buffers increase peptide and protein identification and reduce incubation times. The results indicate that a simultaneous and short, high temperature reduction and alkylation reaction using Tris(2‐carboxyethyl)phosphine hydrochloride and chloroacetamide with a subsequent gel wash improve protein identification and sequence coverage, and diminish peptide side reactions. Additionally, use of 4‐(2‐hydroxyethyl)piperazine‐1‐ethanesulfonic acid buffer allows a significant reduction in the digestion time improving trypsin performance and increasing the peptide recovery. The updates of the in‐gel digestion protocol described here are efficient and offer flexibility to be incorporated in any proteomic laboratory.

## 1 Introduction

Tryptic in‐gel digestion is well established as an efficient and simple method to prepare proteins for identification and quantification by MS.^1^, ^2^ The gel delivers excellent results when mass separation is required or compounds incompatible with MS cannot be excluded from protein extraction protocols.^3^, ^4^ Previous efforts to reduce incubation times have focused on the tryptic digestion step while maintaining efficiency by adding additives or increasing temperature among others.^2^, ^5^, ^6^ Tryptic digestion methods have been extensively improved over the years.^1^, ^7^, ^8^ Recently, an investigation on reducing and alkylating reagents increased the number of proteins identified and protein sequence coverage by replacing the widely used DTT and iodoacetamide (IAA) reagents.^9^

Our work collates recent advances in tryptic digestion to create beneficial updates of the in‐gel digestion protocol.^9^, ^10^ We show superior protein identification and sequence coverage with reduced side reactions and handling time, when compared with the basic approach. We tested the reduction and alkylation of proteins using Tris(2‐carboxyethyl)phosphine hydrochloride (TCEP) and chloroacetamide (CAA) simultaneously at lower concentrations, using a high temperature and shorter incubation time. To eliminate unwanted side reactions, a wash step prior to tryptic digestion was added. The trypsin digestion was optimisedusing 4‐(2‐hydroxyethyl) piperazine‐1‐ethanesulfonic acid buffer (HEPES) rather than ammonium bicarbonate (ABC).

## 2 Experimental Section

To generate test samples, 50 μg of HeLa protein extract was mixed in a 6:1 (v/v) ratio with sample loading buffer (0.375 m Tris‐HCl pH 6.8, 12% SDS, 60% Glycerol, 0.6 m DTT, and 0.06% bromophenol blue; Sigma) and incubated at 95 °C for 5 min. The protein extract was divided into 18 samples, distributed in six different experimental conditions with three replicates each. All samples were run in 10% SDS‐PAGE mini‐gels until the dye front was 1 cm from the bottom for higher reproducibility. (Note: the gels can be run for shorter time to avoid processing large amounts of sample and introduce contaminants). The gels were washed with deionised water and stained with Coomassie protein stain (Expedeon) for 15 min (Figure S1, Supporting Information). Each lane was cut into three equal pieces and each piece was cut into cubes of ≈1 mm^2^. If smaller gel pieces are generated the peptides samples must be filtrated (Corning Costar Spin‐X centrifuge tube filters, cellulose acetate membrane, pore size 0.22 μm) to avoid nano‐column or injector blockage. Gel cubes from the three gel pieces were transferred to three different 1.5 mL tubes (Eppendorf) and destained twice using 50% ethanol (Fisher‐Scientific) in 50 mm ABC (Fluka) at 22 °C, for 15 min and dehydrated with 100% ethanol for additional 5 min, with shaking. (Ethanol was preferred over acetonitrile due to its lower toxicity and environmental impact^11^). The six groups were treated with different methods, and all solutions were prepared fresh and shaken at ≈650 rpm during treatment (Table 1): Method 1 ‘the basic’: dehydrated gel pieces were reduced with 10 mm DTT (Sigma) at 56 °C for 30 min. Alkylation was conducted by replacing the DTT solution with 55 mm IAA (Sigma) and incubated at 22 °C for 20 min in the dark followed by washing with 50% ethanol in 50 mm ABC at 22 °C, for 15 min and dehydrated with 100% ethanol for 5 min . The gel pieces were hydrated with minimum volume required of 2.5 ng μL^–1^ of trypsin (Promega) in 50 mm ABC solution, pH 8 for 1 h at room temperature, topped up with ABC solution until the gel pieces were covered and incubated overnight at 37 °C. Peptides were extracted from gel pieces with consecutive incubations: twice with 25% ACN (Fisher Scientific) with 5 min sonication in a water bath; 100% ACN with 5 min sonication. Supernatants were combined in a fresh vial, dried using a vacuum centrifuge at 50 °C, resuspended in 50 μL of 2% ACN and 0.1% TFA (Fluka) and sonicated in a water bath for 5 min. Methods 2–6 were accumulative variations of method 1 until the final updated method 6 is achieved. Method 2 was the same as method 1 except IAA was replaced by 55 mm CAA (Sigma). Method 3 was the same as method 2 except reduction and alkylation were performed simultaneously with a solution of 10 mm TCEP (Sigma) and 40 mm CAA at room temperature for 20 min. Method 4 was the same as method 3 except reduction/alkylation reaction was incubated at 70 °C for 5 min. Method 5 was the same as method 4 but the tryptic protein digestion was performed for 4 h. Method 6 was the same as method 5 but the ABC buffer was replaced by 50 mm HEPES pH 8.5 (Alfa Aesar).

LC–MS/MS analysis was performed using an Ultimate 3000‐RSLCnano system (Dionex) and an Orbitrap‐Fusion (Thermo‐Scientific). Twenty microliters of sample with 1 μg of peptides, was loaded on an Acclaim‐PepMap μprecolumn (Thermo‐Scientific, 300 μm id × 5 mm length, 5 μm particle size, 100 Å pore size) equilibrated in 2% ACN and 0.1% TFA, for 8 min at 10 μL min^–1^ with an analytical column Acclaim PepMap RSLC (Thermo‐Scientific, 75 μm id × 50 cm, 2 μm, 100 Å). Mobile phase A was of 0.1% formic acid and mobile phase B was ACN containing 0.1% formic acid. Peptides were eluted at 250 nL min^–1^ by increasing the mobile phase B from 8% B to 25% over 90 min, then 35% B over 9 min followed by 90% B for 3 min and a 15 min re‐equilibration at 4% B. To avoid cross‐contamination between samples, a minimum of two washes of 30 min each was run between samples. We recommend loading a maximum of 1 μg of total peptides per injection for complex mixtures or 20–50 ng of peptides for single protein bands to avoid strong cross‐contamination. If higher amounts are injected, additional washes will be necessary. MS data was acquired with Xcalibur v3.0.63 (Thermo‐Scientific). Electrospray used a static Nanospray‐Flex with a stainless steel emitter OD 1/32’ in positive mode at 2.1 kV (Thermo‐Scientific). MS survey scans from 375 to 1575 *m/z*, with a 2 × 10^5^ ion count target, maximum injection time of 150 ms, and resolution of 120 000 at 200 *m/z*, acquired in profile mode were performed in the Orbitrap analyser. Data dependent mode selected the most abundant precursor ions possible in 2 s cycle time followed by 45 s exclusion and ions were isolated in the quadrupole with a 1.2 *m/z* window. MS/MS scans were performed in the ion trap in rapid mode with ion count target of 2 × 10^4^ and maximum injection time of 200 ms and acquired in centroid mode. Precursor ions were fragmented with higher energy C‐trap dissociation (HCD), normalised collision energy of 33% and fixed first mass of 120 *m/z*. Performance of the LC–MS was controlled by running HeLa lysates quality controls (Pierce, ThermoFisher) before and after the experiments. Thermo‐Scientific raw files were analyzed using MaxQuant software v1.6.0.16^12^ against the UniProtKB Human database (UP000005640, 71 785 entries, release March 2017). Peptide sequences were assigned to MS/MS spectra using the following parameters: cysteine carbamidomethylation as a fixed modification and protein N‐terminal acetylation and methionine oxidations as variable modifications. Two separate searches were performed: a) adding peptide N‐terminal acetylation as a variable modification b) changing cysteine carbamidomethylation from a fixed to a variable modification. The FDR was set to 0.01 for both proteins and peptides with a minimum length of seven amino acids and was determined by searching a reversed database. Enzyme specificity was trypsin with a maximum of two missed cleavages. Peptide identification was performed with an initial precursor mass deviation of 7 ppm and a fragment mass deviation of 20 ppm. The MaxQuant feature ‘match between runs’ was enabled only within experimental replicates. Label‐free protein quantification (LFQ) was calculated if a minimum of two peptides was compared between different samples. Data processing was performed using the Perseus module of MaxQuant v1.6.0.16.^13^ Proteins identified by MaxQuant as “Reverse” or false positives, “Potential contaminant” or common contaminant proteins and “Only identified by site” or proteins identified with only one modified peptide were discarded. Only protein groups identified with at least two assigned peptides were accepted and LFQ intensities were log2 transformed. The dataset has been deposited to the ProteomeXchange Consortium^14^ with the dataset identifier PXD009600. This work was MIAPE‐compliant.^15^

## 3 Results and Discussion

**Figure 1:**
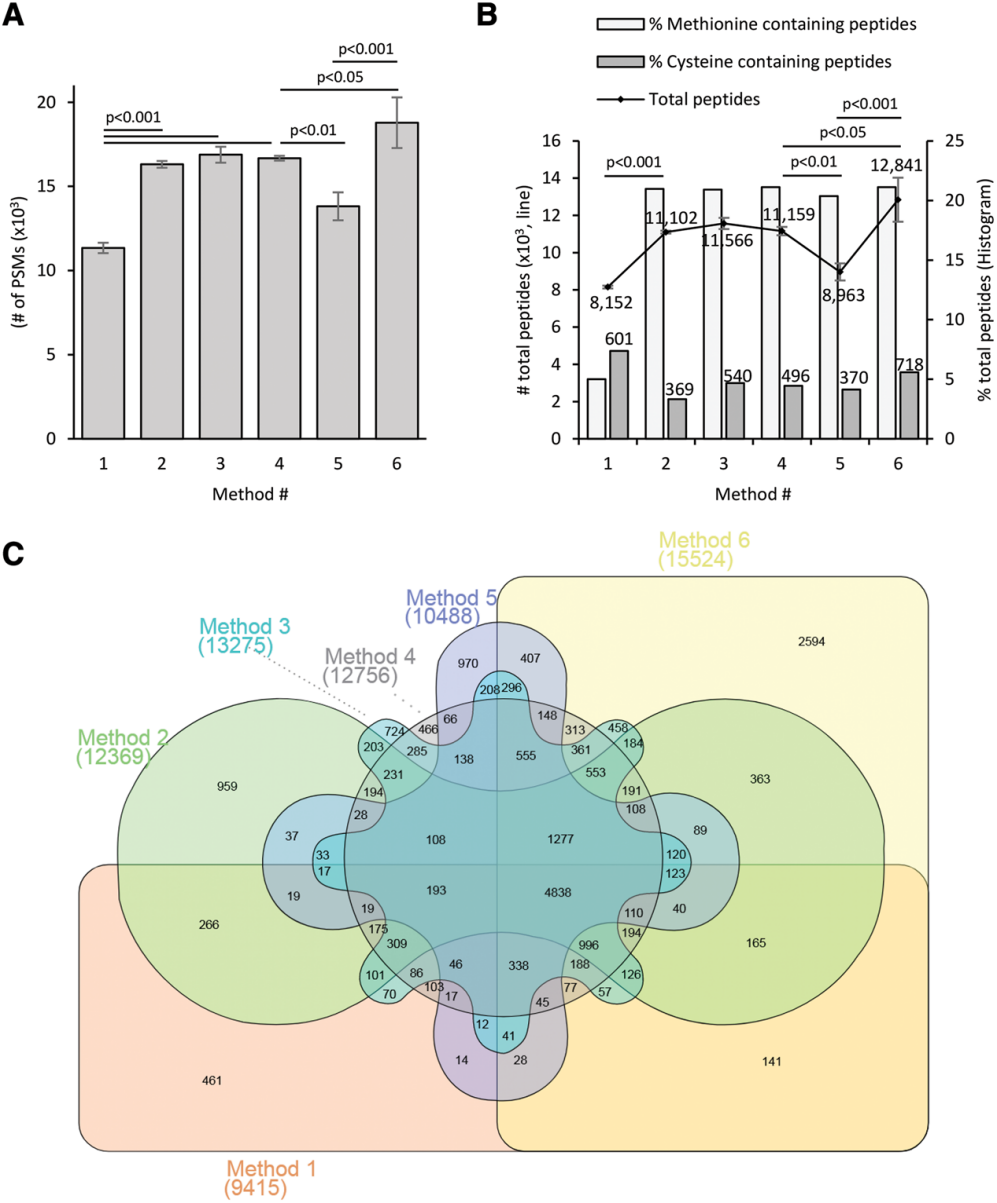
Peptide analysis. A) Bar chart illustrating the number of peptide spectral matches (PMSs; *n* = 3) Bars represents the mean ± SD. Statistical analysis was carried out using GraphPad Prism software (GraphPad Software, Inc.) and *p*‐value was calculated according to the ANOVA using Tukey for multiple comparisons. B) Line represents the mean ± SD of number of common peptides identified and quantified (*n* = 3, primary axe). The associated numbers are the mean values. Statistical analysis was carried out using GraphPad Prism software (GraphPad Software, Inc.) and *p*‐value was calculated according to the ANOVA using Tukey for multiple comparisons. Bars indicate the% of methionine or cysteine containing peptides detected. Top numbers indicate the mean of sum of cysteine peptides (*n* = 3). C) Venn diagram comparing the sum of all identified and quantified peptides (*n* = 3) from methods 1–6. Number between brackets represents the total number of peptides identified and quantified. Venn diagram was created using InteractiveVenn web tool.^18^

**Figure 2:**
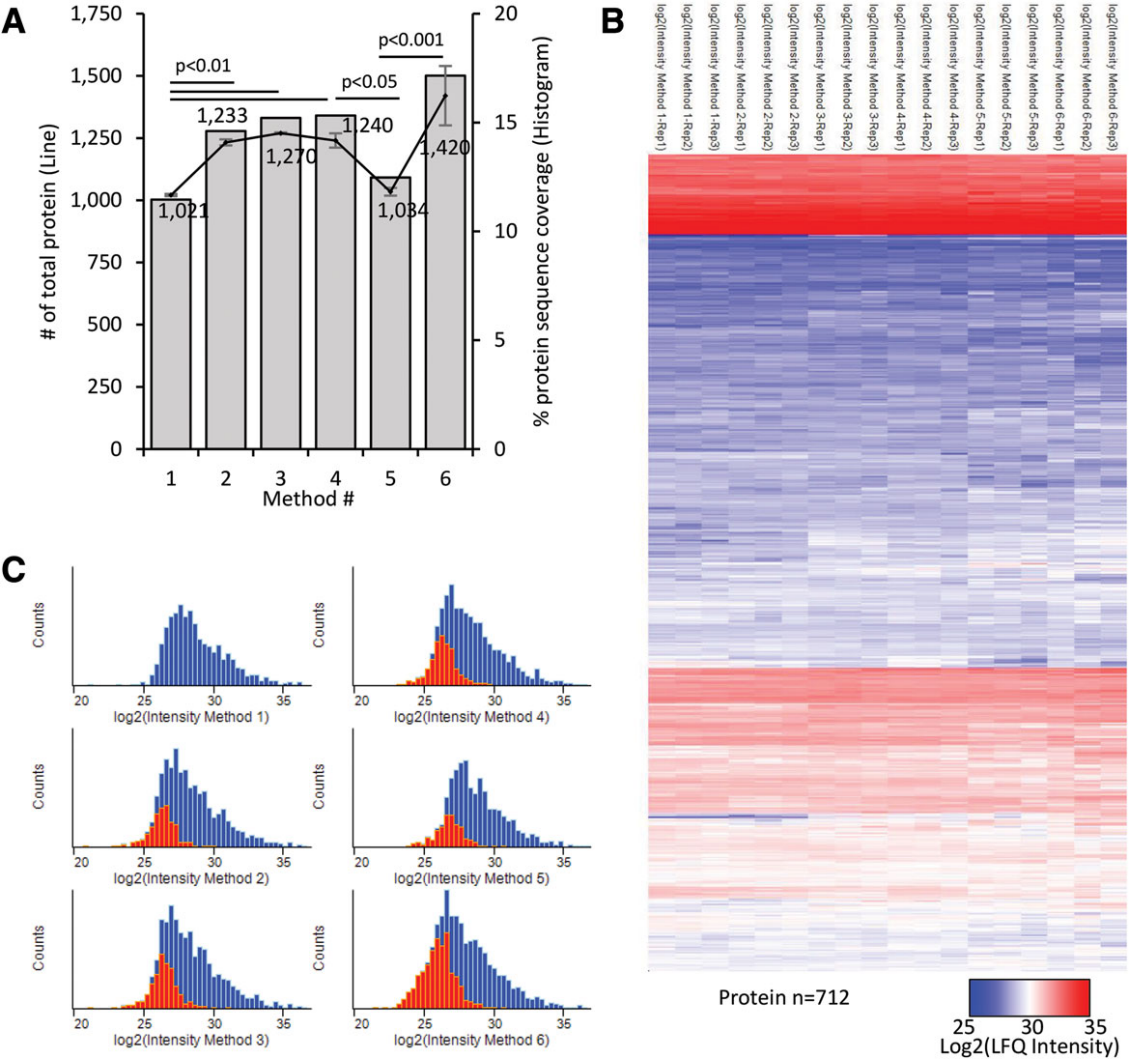
Protein analysis. A) Line represents the mean ± SD of number of common proteins identified and quantified (*n* = 3, primary axe). The associated numbers are the mean values. Bars indicate the mean% of protein sequence coverage. Statistical analysis was carried out using GraphPad Prism software (GraphPad Software, Inc.) and *p*‐value was calculated according to the ANOVA using Tukey for multiple comparisons. B) Hierarchical clustering (based on average Euclidean distance) and heat map (colors based on log_2_ (LFQ‐intensity)) of the protein intensities calculated for the 712 common proteins detected in all methods (*n* = 18). C) Frequency plot of protein LFQ intensities for all methods (blue bars) and the proteins newly identified in comparison with the basic method1 (red bars).

Our objective was to evaluate an updated in‐gel digestion protocol (method 6) by incorporating recent advances in sample preparation (Table 1). By replacing the common alkylation reagent IAA (method 1) with CAA (methods 2–6) the number of identified MS2 fragmentation scans increased while the number of scans triggered remained the same, similar to the extensive investigation published by Muller and Winter.^9^ CAA methods gave higher peptide identifications and fewer side reactions, as determined by number of identified methionine containing peptides (Figure 1 and Figure 2, Supporting Information). Despite the higher peptide identification and over 98% of cysteine alkylation efficiencies, (Figure 2B, Supporting Information), CAA alkylated samples identified only 65% of the cysteine containing peptides detected with IAA under similar conditions (methods 1–2). This is probably due to the higher reactivity of iodine compared to chlorine. TCEP and CAA can be incubated at the same time with the proteins^16^ for efficient reduction and alkylation, as suggested by Winter and Muller.^9^ Therefore, we tested simultaneous addition of TCEP and CAA (method 3) with reduced sample handling and recovered 89% of the cysteine peptides detected with IAA. The updated method 6 showed more cysteine peptides identified (119%) than the basic method (Figure 2B, Supporting Information). The addition of TCEP increased the number of identified tryptic miss‐cleaved peptides by 7% compared to the DTT methods (methods 1–3) and that difference was reduced to 3% by heating the TCEP/CAA reaction at 70 °C for 5 min (method 4) while reducing significantly the incubation time, as previously described for DTT^17^ (Figure 2C, Supporting Information). However, temperatures above 80 °C resulted in overall worse results (data not shown). An additional gel wash included after the reduction/alkylation reaction reduced peptide N‐terminus acetylation to 1.5% of the total peptides detected (data not shown) which was the most important off site alkylation identified previously.^9^ Replacing the ABC digestion buffer with HEPES (method 6), permitted a shorter incubation time and provided the best peptide coverage (Figure 1 and Figure 2, Supporting Information).

**Table 1:** Overview of the sample preparation and digestion conditions for the basic in‐gel digestion (Method1) and updated protocols (methods 2–6)

Protocol step	Conditions	Method					
		1	2	3	4	5	6
Distain	Gel distain	✓	✓	✓	✓	✓	✓
	50% and 100% ethanol						
Reduction	10 mM DTT	✓	✓				
and alkylation	56 °C, 30 min						
	55 mM IAA	✓					
	21 °C, 20 min						
	55 mM CAA		✓				
	21 °C, 20 min						
	10 mM TCEP and 40 mM CAA			✓			
	21 °C, 20 min						
	10 mM TCEP and 40 mM CAA				✓	✓	✓
	70 °C, 5 min						
Clean up	Gel wash	✓	✓	✓	✓	✓	✓
	50% and 100% ethanol						
Tryptic digestion	Tryptic digestion in 50 mM ABC	✓	✓	✓	✓		
	37 °C, over night						
	Tryptic digestion in 50 mM ABC					✓	
	37 °C, 4 h						
	Tryptic digestion in 50 mM HEPES						✓
	37 °C, 4 h						
Peptide extraction	Peptide extraction,	✓	✓	✓	✓	✓	✓
	25% and 100% ACN						

Consistent with our observations at peptide level, the total number of proteins identified, and their sequence coverage, improved when IAA was replaced by CAA, DTT by TCEP, and ABC by HEPES (Figure 2A). Regardless of the method used, the calculated protein LFQ intensities showed high reproducibility within replicates and methods, with Pearson correlation values over 0.93 (Figure 2B and Figure 3, Supporting Information). The exclusive population of proteins identified by our updated method contributed to increase in the dynamic range of proteins detected in the low intensity range of the total LFQ intensity distributions (Figure 2C). Additionally, we tested where our methods presented bias protein identification by comparative gene ontology category enrichment analysis and we did not find any difference (data not shown).

## 4 Conclusion

In conclusion, we have shown that our updated in‐gel digestion protocol (method 6) allows the identification of more peptides and proteins by reducing the side reactions and increasing sensitivity when compared with the basic approach. The increased identification of peptides is important to confidently identify and quantify proteins and also for investigations aiming for maximum protein sequence coverage or identification of posttranslational modifications. Additionally, the updated protocol reduces sample handling and incubation times; decreasing the probability of contamination and making affordable sample preparation and MS analysis in 1 day.

## Conflict of Interest

The authors declare no conflict of interest.

## Supporting information

figureS1

figureS2

figureS3
