# Supplementary material for: Updates of the In‐Gel Digestion Method for Protein Analysis by Mass Spectrometry
Source: Proteomics. 2018 Nov 25;18(23):1800236. doi: 10.1002/pmic.201800236 (PMC6492177; doi:10.1002/pmic.201800236)
Supplement: Supplementary file 1 — figureS1 [file PMIC-18-na-s001.pptx]

## Slide 1
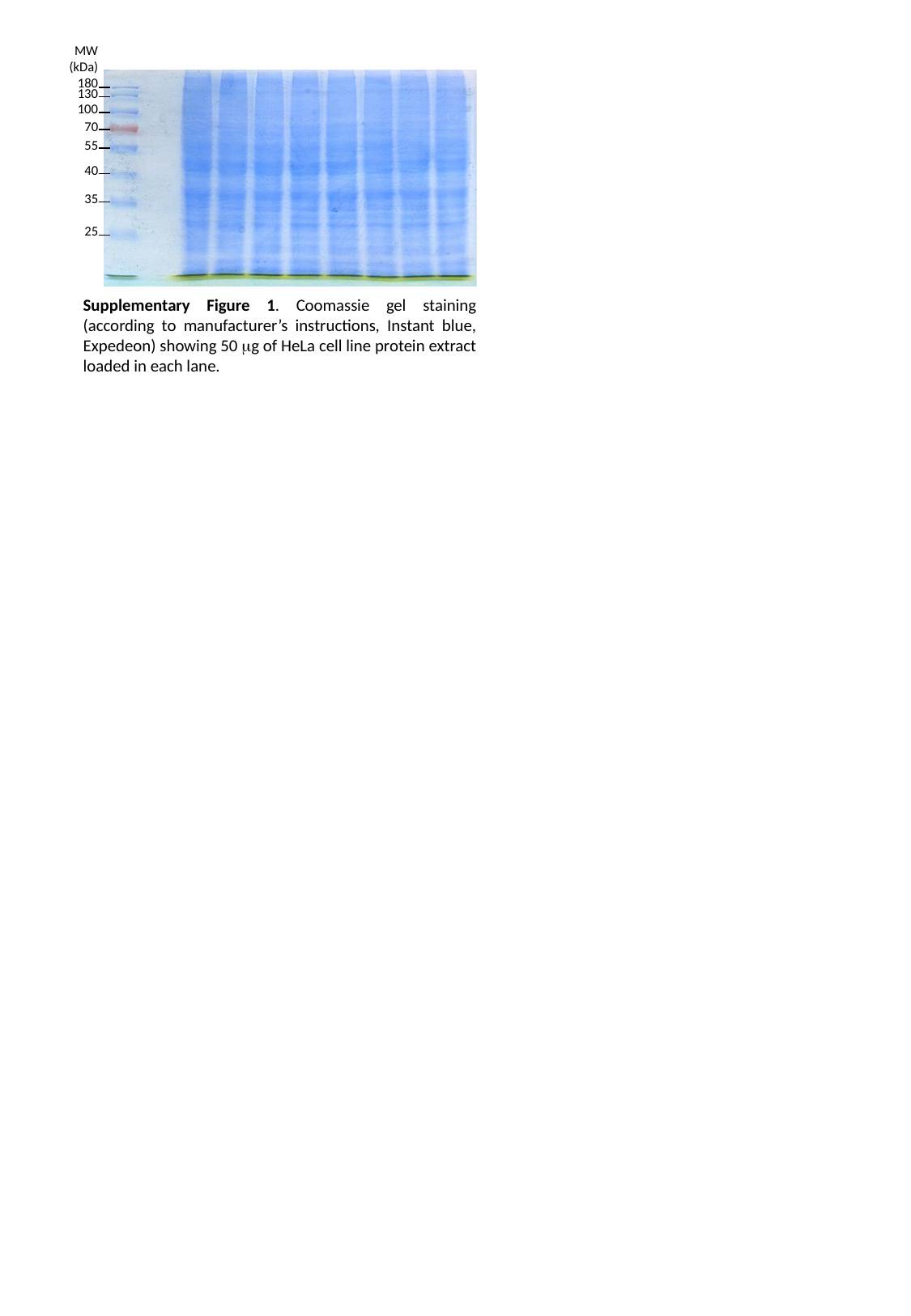

MW
(kDa)
180
130
100
70
55
40
35
25
Supplementary Figure 1. Coomassie gel staining (according to manufacturer’s instructions, Instant blue, Expedeon) showing 50 μg of HeLa cell line protein extract loaded in each lane.
