# Supplementary material for: Updates of the In‐Gel Digestion Method for Protein Analysis by Mass Spectrometry
Source: Proteomics. 2018 Nov 25;18(23):1800236. doi: 10.1002/pmic.201800236 (PMC6492177; doi:10.1002/pmic.201800236)
Supplement: Supplementary file 2 — figureS2 [file PMIC-18-na-s002.pptx]

## Slide 1
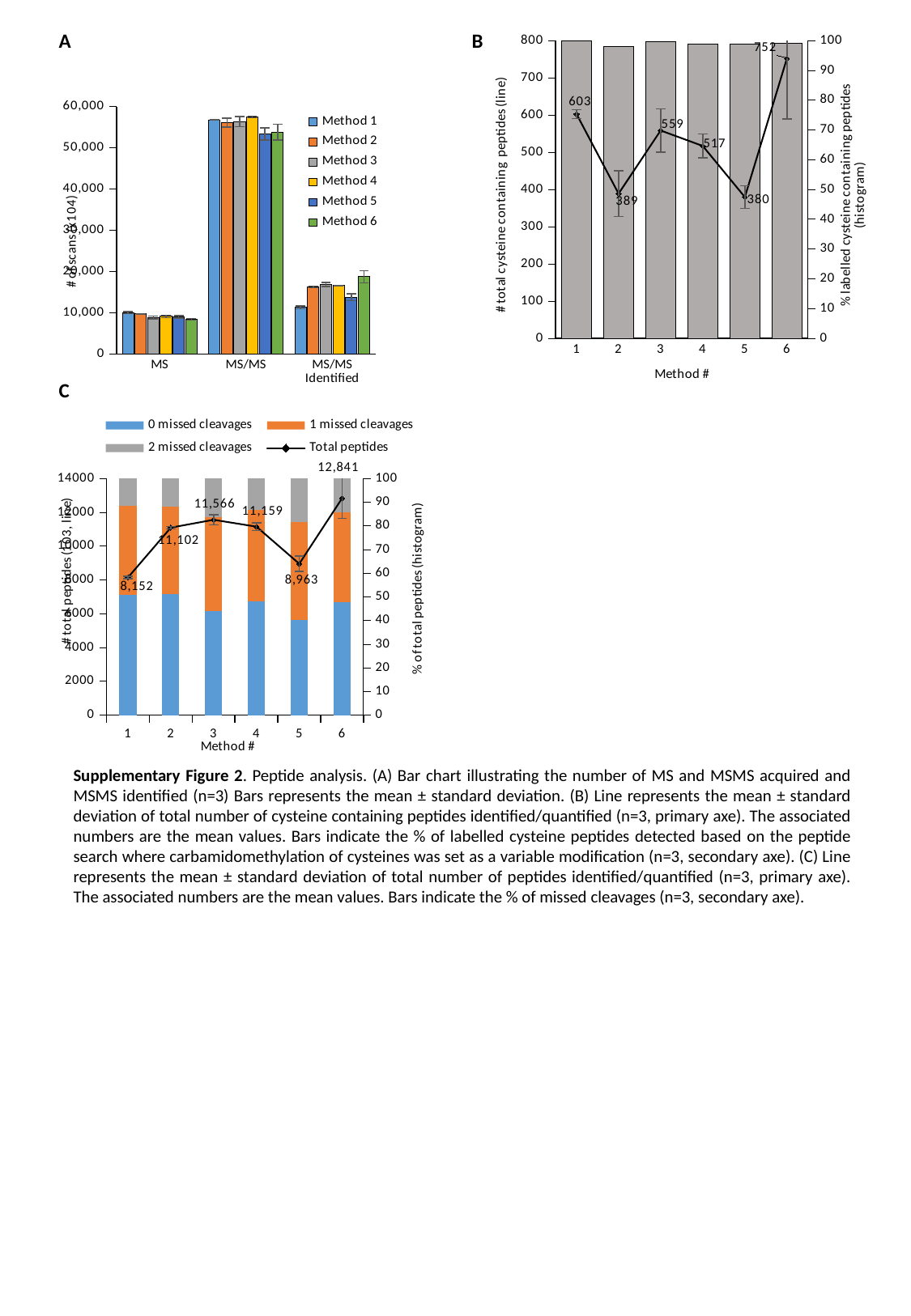

A
B
### Chart
| Category | % modified c peptides | Total cysteine peptides |
|---|---|---|
### Chart
| Category | Method 1 | Method 2 | Method 3 | Method 4 | Method 5 | Method 6 |
|---|---|---|---|---|---|---|
| MS | 10083.666666666666 | 9815.333333333334 | 8893.0 | 9196.0 | 9078.0 | 8493.333333333334 |
| MS/MS | 56823.333333333336 | 56187.0 | 56422.666666666664 | 57578.666666666664 | 53396.0 | 53824.666666666664 |
| MS/MS Identified | 11343.0 | 16308.666666666666 | 16884.0 | 16671.666666666668 | 13818.0 | 18783.333333333332 |C
### Chart
| Category | 0 missed cleavages | 1 missed cleavages | 2 missed cleavages | Total peptides |
|---|---|---|---|---|
| 1 | 50.983358547655065 | 37.45348979842173 | 11.563151653923212 | 8152.333333333333 |
| 2 | 51.05239453535506 | 37.04849121753491 | 11.899114247110044 | 11101.666666666666 |
| 3 | 43.865463872957314 | 39.91411361212785 | 16.220422514914834 | 11565.666666666666 |
| 4 | 48.1150675110527 | 38.51415939777752 | 13.370773091169793 | 11158.666666666666 |
| 5 | 40.27519523986612 | 41.4020081814801 | 18.322796578653772 | 8963.333333333334 |
| 6 | 47.700134980791184 | 38.158031357076105 | 14.141833662132695 | 12841.333333333334 |Supplementary Figure 2. Peptide analysis. (A) Bar chart illustrating the number of MS and MSMS acquired and MSMS identified (n=3) Bars represents the mean ± standard deviation. (B) Line represents the mean ± standard deviation of total number of cysteine containing peptides identified/quantified (n=3, primary axe). The associated numbers are the mean values. Bars indicate the % of labelled cysteine peptides detected based on the peptide search where carbamidomethylation of cysteines was set as a variable modification (n=3, secondary axe). (C) Line represents the mean ± standard deviation of total number of peptides identified/quantified (n=3, primary axe). The associated numbers are the mean values. Bars indicate the % of missed cleavages (n=3, secondary axe).
