# Supplementary material for: Updates of the In‐Gel Digestion Method for Protein Analysis by Mass Spectrometry
Source: Proteomics. 2018 Nov 25;18(23):1800236. doi: 10.1002/pmic.201800236 (PMC6492177; doi:10.1002/pmic.201800236)
Supplement: Supplementary file 3 — figureS3 [file PMIC-18-na-s003.pdf]

PROTEOMICS

Supporting Information
for Proteomics
DOI 10.1002/pmic.201800236

Jennifer K. Goodman, Cleidiane G. Zampronio, Alexandra M. E. Jones and Juan R. Hernandez-Fernaud

Updates of the In-Gel Digestion Method for Protein Analysis by Mass Spectrometry

© 2018 University of Warwick. *Proteomics* Published by WILEY-VCH Verlag GmbH & Co. KGaA, Weinheim.**www.proteomics-journal.com**

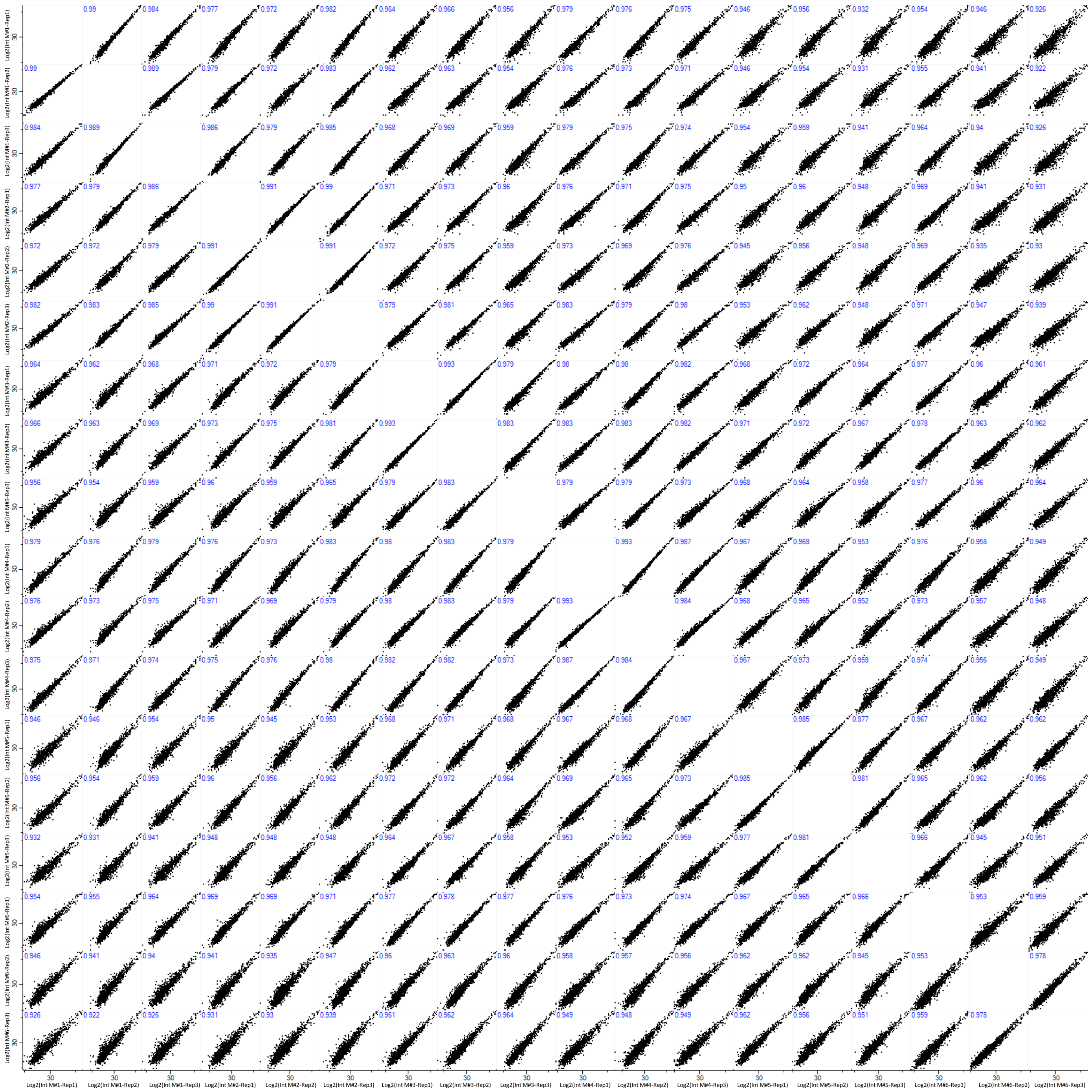

Supplementary Figure 4. Protein analysis. Pearson correlation coefficient analysis of the Label Free Quantification intensities (LFQ; log2) which shows high correlation among methods (M#1-6) and their replicates (Rep1-3). The number in the top left corner of each plot represents the calculated Pearson correlation value.
